# Supplementary material for: The Isolation and Characterization of Perlucin in Pacific Abalone, Haliotis discus hannai: A Shell Morphogenic Protein with Potential Responses to Thermal Stress and Starvation
Source: Biology (Basel). 2024 Nov 18;13(11):944. doi: 10.3390/biology13110944 (PMC11591584; doi:10.3390/biology13110944)
Supplement: Supplementary file 1 [file biology-13-00944-s001.zip › biology-3286727-supplementary.pdf]

Table S1: Protein sequences used for phylogenetic analysis, multiple sequence alignment, and homology analysis

Common Name	Scientific Name	Gene	GenBank Accession No.
			Protein
Pacific abalone	*Haliotis discus hannai*	*Perlucin*	UTD53617.1
Variously colored abalone	*Haliotis diversicolor*	*Perlucin*	ADD16957.1
Disc abalone	*Haliotis discus discus*	*Perlucin 8*	ABO26597.1
Variously colored abalone	*Haliotis diversicolor*	*Perlucin 6*	AEQ16380.1
Disc abalone	*Haliotis discus discus*	*Perlucin 6*	ABO26595.1
Variously colored abalone	*Haliotis diversicolor*	*Perlucin 4*	AEQ16381.1
Disc abalone	*Haliotis discus discus*	*Perlucin 7*	ABO26596.1
Variously colored abalone	*Haliotis diversicolor*	*Perlucin 1*	AEQ16377.1
Disc abalone	*Haliotis discus discus*	*Perlucin 1*	ABO26590.1
Disc abalone	*Haliotis discus discus*	*Perlucin 4*	ABO26593.1
Greenlip abalone	*Haliotis laevigata*	*Perlucin C*	CBK19535.1
Greenlip abalone	*Haliotis laevigata*	*Perlucin B*	CBK19534.1
Greenlip abalone	*Haliotis laevigata*	*Perlucin A2*	CBK19533.1
Greenlip abalone	*Haliotis laevigata*	*Perlucin A*	CBK19532.1
Disc abalone	*Haliotis discus discus*	*Perlucin 5*	ABO26594.1
Disc abalone	*Haliotis discus discus*	*Perlucin 3*	ABO26592.1
Variously colored abalone	*Haliotis diversicolor*	*Perlucin 5*	AEQ16379.1
Bloodfluke planorb	*Biomphalaria glabrata*	*Perlucin*	KAI8777934.1
Ram's horn snails	*Biomphalaria pfeifferi*	*Perlucin*	KAK0067466.1
European physa	*Physella acuta*	*perlucin-like*	XP_059176067.1
Pacific oyster	*Crassostrea gigas*	*Perlucin*	XP_011411987.2
Ornate sap-sucking slug	*Elysia marginata*	*Perlucin*	GFS17464.1
Akoya pearl oyster	*Pinctada fucata*	*Perlucin*	JAS04077.1
Northern quahog	*Mercenaria mercenaria*	*perlucin-like*	XP_045191035.2
Hong Kong oyster	*Crassostrea hongkongensis*	*Perlucin*	URX65653.1
Pacific blue mussel	*Mytilus trossulus*	*perlucin-like*	XP_063399102.1
European physa	*Physella acuta*	*perlucin-like*	XP_059139915.1
Variously colored abalone	*Haliotis diversicolor*	*Perlucin 2*	AEQ16378.1
Disc abalone	*Haliotis discus discus*	*Perlucin 2*	ABO26591.1
White springtail	*Folsomia candida*	*Perlucin*	OXA44722.1
hairy-Back Girdled Springtail	*Orchesella cincta*	*Perlucin*	ODN00705.1
Yaba Nigerian Nail Mosquito	*Anopheles moucheti*	*perlucin-like*	XP_052902878.1

Mosquito	*Anopheles nili*	*perlucin-like*	XP_053677323.1
The malaria mosquito	*Anopheles marshallii*	*perlucin-like*	XP_053667667.1
African malaria mosquito	*Anopheles gambiae*	*Perlucin*	XP_563214.3
Malaya	*Malaya genurostris*	*perlucin-like*	XP_058462524.1
Green mud crab	*Scylla paramamosain*	*perlucin-like*	XP_063872522.1
Nail mosquito	*Anopheles coustani*	*perlucin-like*	XP_058121876.1
Marsh mosquito	*Anopheles cruzii*	*perlucin-like*	XP_052860567.1
Nail mosquito	*Anopheles bellator*	*perlucin-like*	XP_058063422.1
Northern house mosquito	*Culex pipiens pallens*	*perlucin-like*	XP_039451224.1
Southern house mosquito	*Culex quinquefasciatus*	*Perlucin*	XP_038114780.1
Mediterranean fruit fly	*Ceratitis capitata*	*Perlucin*	JAC05710.1
Oriental fruit fly	*Bactrocera dorsalis*	*perlucin*	JAC56050.1
Mountain pine beetle	*Dendroctonus ponderosae*	*perlucin*	XP_048523942.1
House fly	*Musca domestica*	*perlucin-like*	XP_005186594.3
Sweetpotato weevil	*Cylas formicarius*	*perlucin-like*	XP_060524813.1
Western corn rootworm	*Diabrotica virgifera virgifera*	*perlucin*	XP_050513491.1
Stable fly	*Stomoxys calcitrans*	*perlucin-like*	XP_013119384.1
Asian long-horned beetle	*Anoplophora glabripennis*	*perlucin*	XP_018561628.1
Sand fly	*Lutzomyia longipalpis*	*perlucin-like*	XP_055682798.1
Subtropical tamarisk beetle	*Diorhabda sublineata*	*perlucin-like*	XP_056637142.1
Bird cherry-oat aphid	*Rhopalosiphum padi*	*perlucin-like*	XP_060852219.1
Australian bush fly	*Musca vetustissima*	*perlucin-like*	XP_061401395.1
Mexican fruit fly	*Anastrepha ludens*	*perlucin-like*	XP_053963179.1
Melon fly	*Zeugodacus cucurbitae*	*perlucin-like*	XP_011180433.2
Australian sheep blowfly	*Lucilia cuprina*	*perlucin*	KAI8119985.1
Dog fly	*Stomoxys calcitrans*	*perlucin*	XP_013119380.2
Melon fly	*Zeugodacus cucurbitae*	*Perlucin*	JAC96974.1
Solanum fruit fly	*Bactrocera latifrons*	*Perlucin*	JAI49557.1
Pale-Footed Uranotaenia	*Uranotaenia lowii*	*perlucin-like*	XP_055612724.1
Asian tiger mosquito	*Aedes albopictus*	*Perlucin*	XP_029732103.2
Asian tiger mosquito	*Aedes albopictus*	*perlucin-like*	XP_062716354.1

Muggensoort	*Topomyia yanbarensis*	*perlucin-like*	XP_058837790.1
Malaya	*Malaya genurostris*	*perlucin-like*	XP_058461646.1
Yellow fever mosquito	*Aedes aegyptica*	*Perlucin*	XP_001651192.2
Elephant mosquito	*Toxorhynchites rutilus*	*perlucin-like*	XP_055618599.1
Iridescent Paddle Mosquito	*Sabethes cyaneus*	*perlucin-like*	XP_053686829.1
Pitcher plant mosquito	*Wyeomyia smithii*	*perlucin-like*	XP_055536471.1
Freshwater Mussel	*Hyriopsis cumingii*	*Perlucin*	AGI61062.1
Akoya pearl oyster	*Pinctada fucata*	*Perlucin*	JAS04076.1
Pacific blue mussel	*Mytilus trossulus*	*perlucin-like*	XP_063425845.1
Eastern Oyster	*Crassostrea virginica*	*perlucin-like*	XP_022330928.1
Japanese oyster	*Magallana gigas*	*Perlucin*	XP_011455487.3

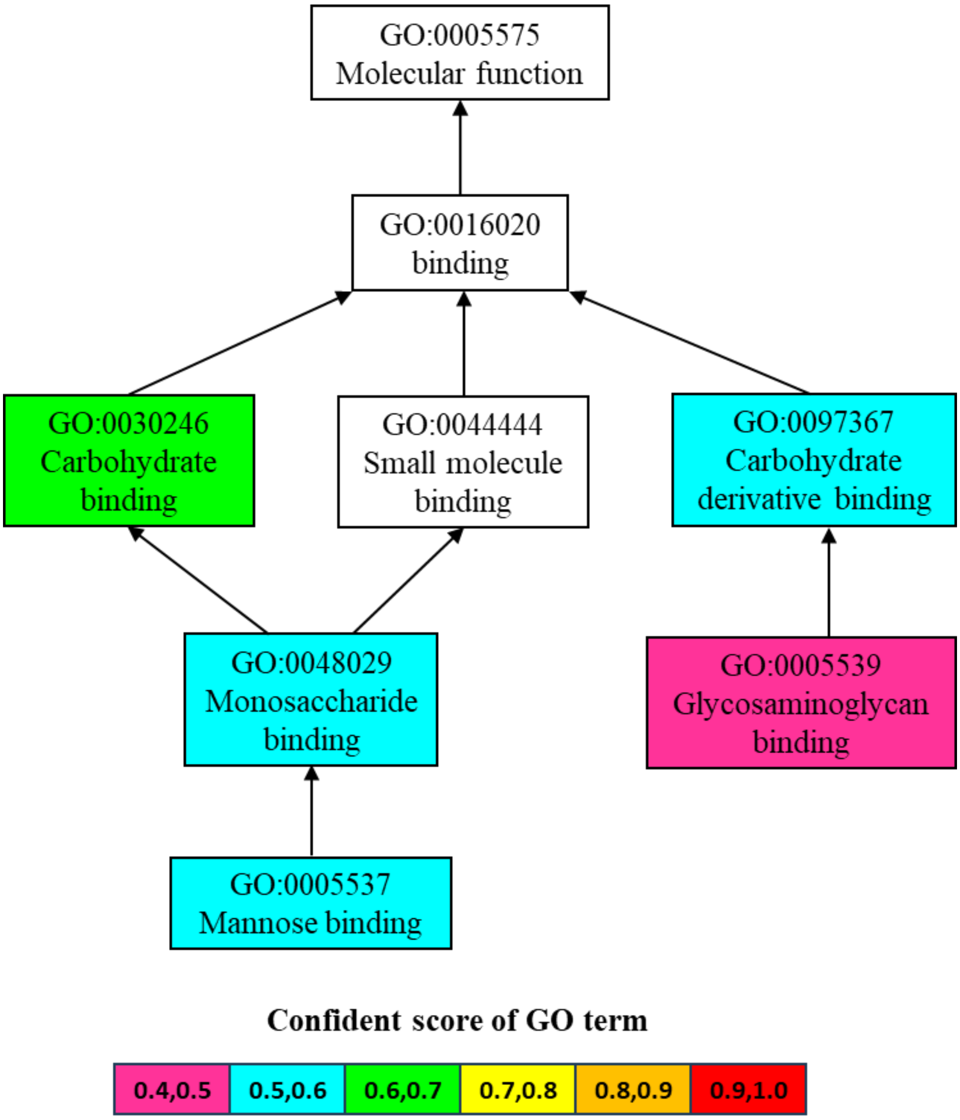

Figure S1. Molecular function (gene ontology) analysis of *Hdh-perlucin* in Pacific abalone. Different colored boxes indicate confidence score for predicting function. High confident score signifies high accuracy.
